# Using neural networks to mine text and predict metabolic traits for thousands of microbes

**DOI:** 10.1371/journal.pcbi.1008757

**Published:** 2021-03-02

**Authors:** Timothy J. Hackmann, Bo Zhang

**Affiliations:** Department of Animal Science, University of California, Davis, United States of America; DAL, CANADA

## Abstract

Microbes can metabolize more chemical compounds than any other group of organisms. As a result, their metabolism is of interest to investigators across biology. Despite the interest, information on metabolism of specific microbes is hard to access. Information is buried in text of books and journals, and investigators have no easy way to extract it out. Here we investigate if neural networks can extract out this information and predict metabolic traits. For proof of concept, we predicted two traits: whether microbes carry one type of metabolism (fermentation) or produce one metabolite (acetate). We collected written descriptions of 7,021 species of bacteria and archaea from *Bergey’s Manual*. We read the descriptions and manually identified (labeled) which species were fermentative or produced acetate. We then trained neural networks to predict these labels. In total, we identified 2,364 species as fermentative, and 1,009 species as also producing acetate. Neural networks could predict which species were fermentative with 97.3% accuracy. Accuracy was even higher (98.6%) when predicting species also producing acetate. Phylogenetic trees of species and their traits confirmed that predictions were accurate. Our approach with neural networks can extract information efficiently and accurately. It paves the way for putting more metabolic traits into databases, providing easy access of information to investigators.

## Author summary

Most information about microbes and their traits is buried in text of books and journals. Investigators who need information on many species are thus doomed to long literature searches. Investigators could avoid this fate, however, if they had a way to extract information from text computationally. We introduce an approach that can extract information with neural networks, a form of machine learning. For proof of concept, we use our approach to predict two metabolic traits for 7,000 species of microbes. This approach was accurate, and it could be used to construct accurate phylogenetic trees of microbes and traits. The work paves the way to large databases of metabolic traits and other information, helping investigators working with big data.

## Introduction

Microbes are everywhere and can metabolize a huge array of chemical compounds. This makes their metabolism important to nutrient cycling in the environment [1–3]. Their metabolism is also important to symbiotic relationships with other organisms [4,5] and for synthetic biologists in the lab [6,7]. As such, information on microbial metabolism is of value to investigators throughout biology.

Despite the value, information on microbial metabolism is hard to access. Books and journals are filled with this information, but it remains buried in text. *Bergey’s Manual of Systematics of Archaea and Bacteria* [8], for example, reports metabolic traits for thousands of microbes, but in the form of long written descriptions. Looking up information for a few species is feasible, but in the era of big data, investigators often need information on many species.

Information on metabolic traits would more useful if extracted from text and summarized in a database. To date, there is no fast and accurate way of extracting this type of information. One method is to employ teams of curators to read articles and extract information manually [9–11]. This method is slow, and information is likely incomplete. Another method is to use machine learning and extract information computationally [12]. This method is fast, but accuracy has not been high enough to be adopted by database curators (see ref. [10]).

The field of machine learning has advanced, and it may now have the accuracy needed to extract metabolic information. Neural networks, one form of machine learning, perform well in extracting other kinds of information from scientific literature [13–17]. When given medical abstracts, for example, neural networks can recognize and extract out names of diseases [14,15]. Their success with other tasks suggests use in extracting information, such as metabolic traits, from microbiology literature.

Here we use neural networks to analyze written descriptions of over 7,000 species of microbes and predict their metabolic traits. For proof of concept, we predicted two traits: whether microbes carried out one type of metabolism (fermentation) or produced one metabolite (acetate). Accuracy in predicting these traits was high (>95%). Our approach paves the way to building large databases of metabolic traits, helping investigators working with big data.

## Results

### Collecting text and labels for thousands of microbes

**Fig 1:**
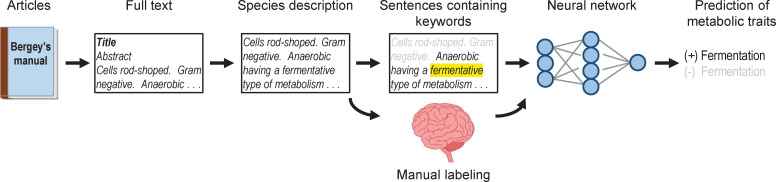
Our approach to predicting metabolic traits with neural networks.

Our general approach to predicting metabolic traits is outlined in Fig 1. We obtained text (written descriptions of microbial species) from *Bergey’s Manual* [8]. From this text, we manually labelled metabolic traits. These labels, along with the written descriptions, served as training data for the network. After training with labels and text, we used the network to predict metabolic traits.

From *Bergey’s Manual* [8], we obtained written descriptions for a total of 7,021 species (see list in S1 Table). To accomplish this, we downloaded the full text of all genus-level articles (n = 1,503). We extracted out species names, then located relevant sections of text for each species. This extraction was an involved process because names and text for each species were scattered through articles (see Methods). We assembled the text into coherent species descriptions.

From these descriptions, we manually labelled species as positive or negative for two metabolic traits. The first trait was general: whether microbes carried out one type of metabolism (fermentation). We searched species descriptions for keyword “ferment”. A total of 4,349 descriptions contained the keyword, and we read these descriptions in full. After reading, we labeled species as positive or negative for the trait. Labels (including justifications) are given in S1 Table. The second trait was more specific: whether fermentative species produced one metabolite (acetate). We searched for keywords (“ferment” plus “acetate” or “acetic”), read matching descriptions (n = 3,987), then labeled species as positive or negative (see S1 Table). Using this approach, we labeled 2,364 species as positive for fermentation, of which 1,009 were also positive for producing acetate. These labels, along with species descriptions, served as training data for the neural network.

### Neural networks accurately predict metabolic traits

After obtaining species descriptions and training data, we trained neural networks and evaluated their performance in predicting metabolic traits. Training was done using TensorFlow [18] as described in the Methods. Evaluations were done with data independent from training.

**Fig 2:**
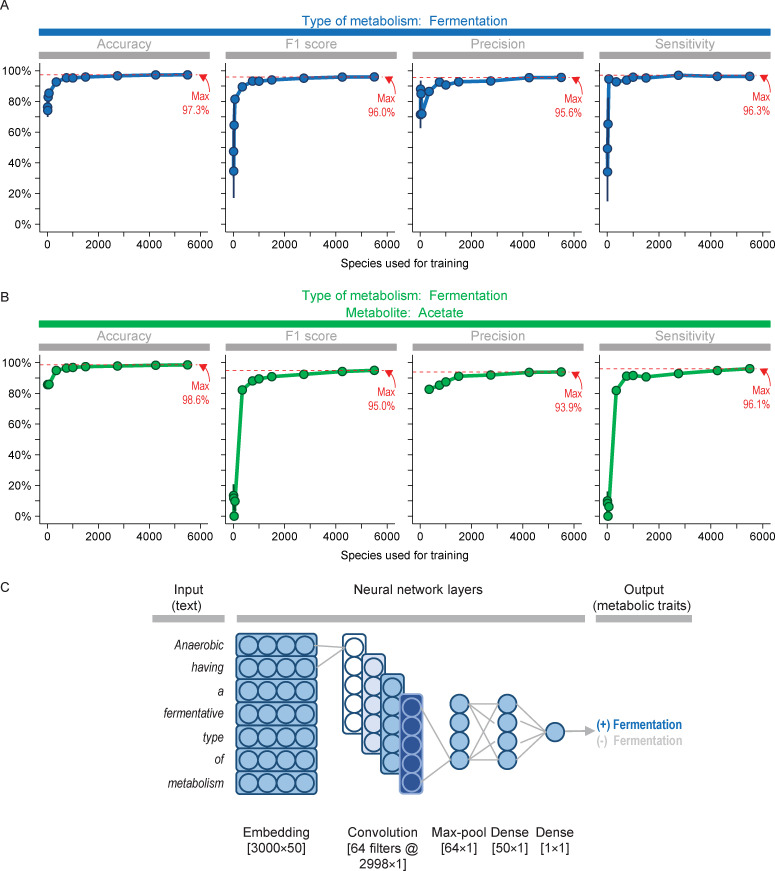
Convolutional neural networks perform well in predicting metabolic traits. (A) Predictions for first trait (fermentative metabolism). (B) Predictions for second trait (acetate production). (C) Architecture of model. Values are means ± SEM of five replicates (independent trainings of the network). Some values for precision are missing because they were undefined (one or more replicates had no false or true positives). For clarity, the number of units depicted in neural network layers is fewer than actual. Units in embedding and hidden dense layers had dropout rate of 0.2.

We found neural networks could predict the first metabolic trait (fermentative metabolism) with high accuracy (Fig 2A). Accuracy increased with the amount of training data, and descriptions for 1,000 species were enough to achieve 95.3% accuracy. Besides high accuracy, predictions from neural networks achieved high F1 score, precision, and sensitivity (Fig 2A). Example predictions (from one training with data for 1,000 species) are shown in S1 Table.

Neural networks achieved similarly high accuracy when predicting the second trait (acetate production) (Fig 2B). In sum, neural networks could accurately predict both general and specific traits.

Few computational resources were required to train the networks and predict metabolic traits. When descriptions for 1,000 species were used, for example, these steps required less than 1 min and 1.5 GiB of memory to complete (S1 Fig). This result shows that networks were not only accurate, but easy to deploy.

**Fig 3:**
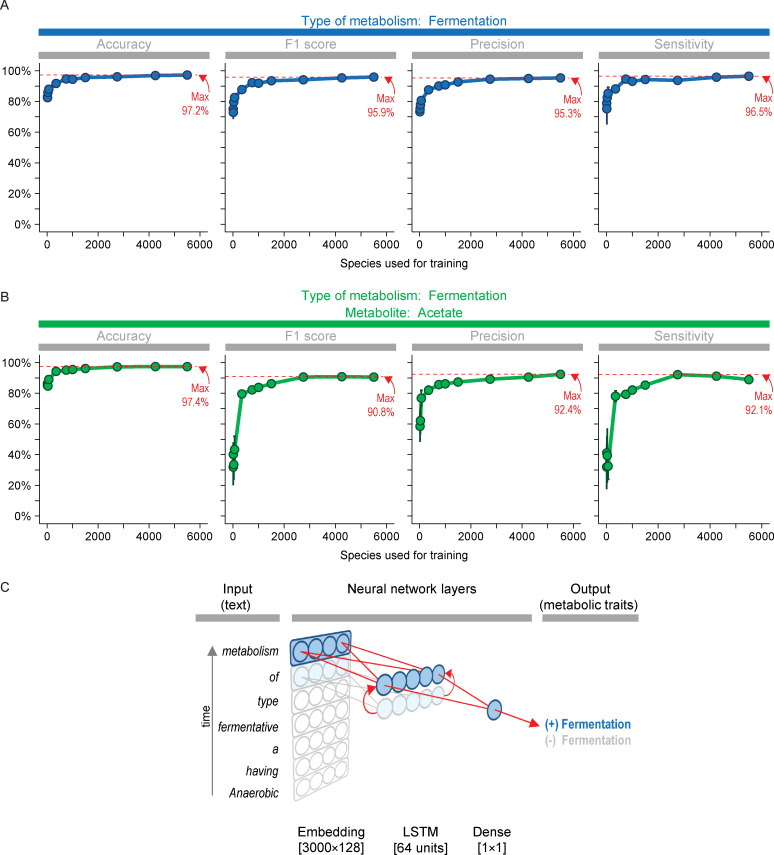
Long short-term memory (LSTM) networks also perform well in predicting traits, though not at the same level as convolutional neural networks. As Fig 2, except type of network is LSTM. Units in the LSTM layer had a dropout rate of 0.2.

Results above are for the best type of neural network. This type was a convolutional network with architecture shown in shown in Fig 2C. We tried other types of networks, and a long short-term memory (LSTM) network also performed well (Fig 3). When little training data was used, its performance equaled or even exceeded that of the convolutional network. However, its performance was overtaken by the convolutional network when using more training data.

Performance depended not only on the type of network, but also how the text was processed before inputted into the network. The highest performance (shown in Fig 2) was achieved when the text (species description) was winnowed down to sentences matching key words (e.g., “ferment”). If the full text was used, much more training data was needed (S2 Fig), and performance was never as high. We have thus taken several steps to optimize the network and ensure predictions of metabolic traits are as high as possible.

### Predictions from neural networks yield accurate phylogenetic trees

**Fig 4:**
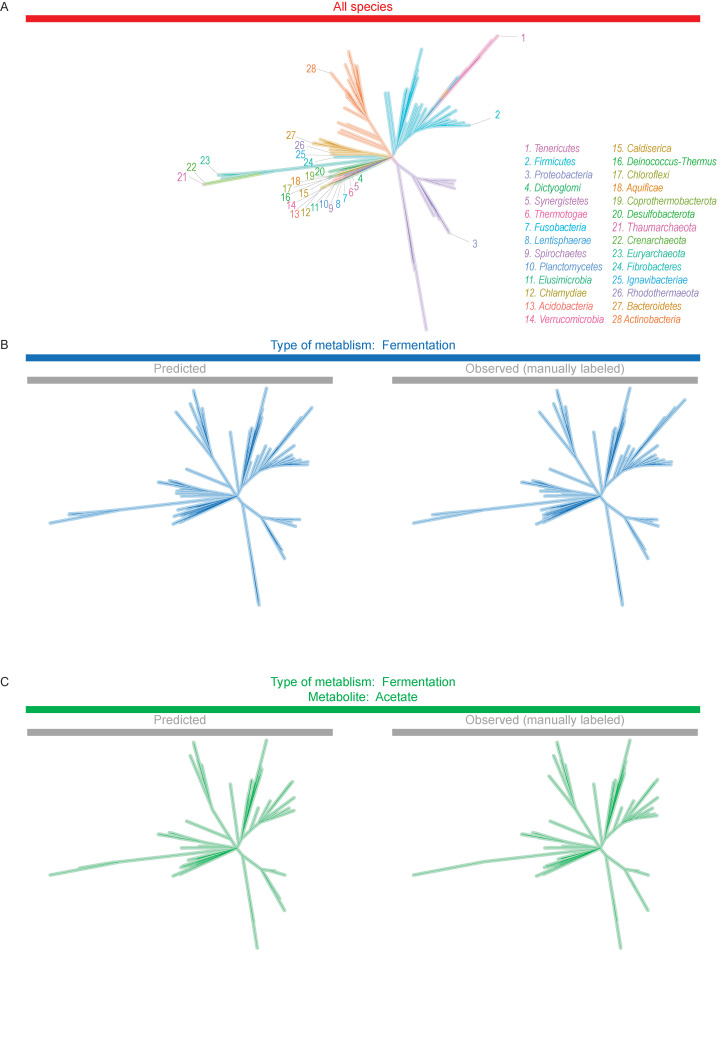
Predictions of neural networks lead to accurate phylogenetic trees. (A) All species in *Bergey’s Manual* [8] with available sequences. (B) Species with first trait (fermentative metabolism). (C) Species with second trait (acetate production). To generate the predicted tree, traits were predicted with a convolutional neural network and training data for 1,000 species. The predicted and observed trees shown are representative of five replicates (independent trainings of the network). Trees were constructed with concatenated ribosomal protein sequences as described in Methods.

We evaluated neural networks further by constructing phylogenetic trees with their predictions. First, we made a phylogenetic tree of all species in *Bergey’s Manual* [8] (Fig 4A). Next, we highlighted species predicted to have the first trait (fermentative metabolism) (Fig 4B). In a separate tree, we highlighted species observed (manually labeled) to have the trait (Fig 4B). These predicted and observed trees appeared similar, meaning predicted species were similar to those observed as having it. Further, the UniFrac distance between predicted and observed trees was small (S3 Fig), confirming that they are similar. We found similar agreement between trees for the second trait (acetate production) (Figs 4C and S3). For both traits, we used training data for 1,000 species.

In sum, predictions from neural networks were not just accurate in a statistical sense. They produced phylogenetic trees that were close to the actual ones, showing they are accurate biologically.

### Databases reporting metabolic traits are incomplete

Some information on metabolic traits can already be found in databases, but it is not clear how complete it is. Our work identified two traits for a number of species, and so it can help assess how complete are these databases for these two traits.

**Fig 5:**
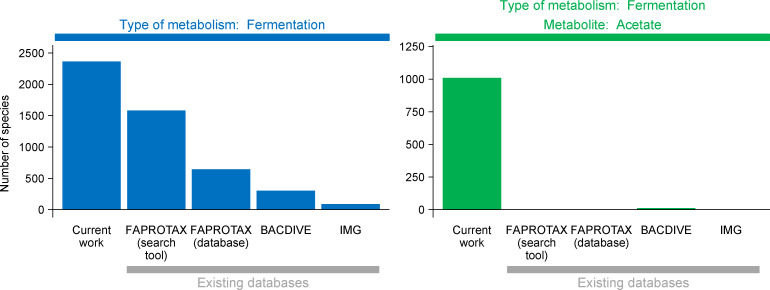
In comparison to the current work, existing databases reporting metabolic traits are incomplete. Species in FAPROTAX were counted in two different ways. First, we used it strictly as a database; we counted species in the database packaged with the tool. Second, we used FAPROTAX as a search tool. We inputted into FAPROTAX the n = 7,021 species from *Bergey’s Manual* used in the current work. See Methods for more details on FAPROTAX and other databases.

As mentioned, our work identified 2,364 species that carried out fermentation. By comparison, the best database identified 1,584 species, or 67% of our number (Fig 5). For species that also produce acetate, the best database identified 1.2% of our number. Some databases (e.g., FAPROTAX) were not designed to identify species that produce acetate, explaining the low completeness for this trait.

Our own numbers of species are incomplete, and thus the situation is worse than it first appears. We obtained descriptions for 7,021 species, yet the total number of species validly published in the literature is 20,038 (see ref. [19]) and increasing by 600 per year [20]. In total, our results suggest that databases reporting the two metabolic traits we investigated are incomplete.

### Negative labels for traits are reliable

When we labeled a species as negative for fermentation, often it was because the species description made no mention of this trait (see S1 Table). It is possible that some species were fermentative, but descriptions in *Bergey’s Manual* were incomplete. To see if this was a problem, we compared descriptions from *Bergey’s Manual* with those from the primary literature (journal articles). We did so for 64 species of fermentative bacteria from the cattle rumen (S2 Table), many of which we study in our lab [21–24].

**Fig 6:**
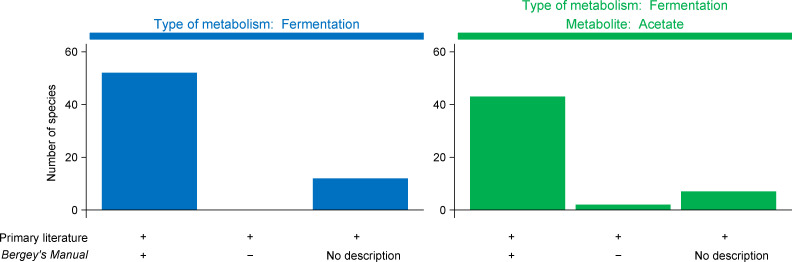
Species descriptions in *Bergey’s Manual* closely agree with the primary literature for the two traits we examined. See S4 Table for details.

We found that descriptions from *Bergey’s Manual* and the primary literature agreed closely (Fig 6 and S4 Table). If a description was available in *Bergey’s Manual*, it always reported the species as positive for fermentation. These results suggest that species descriptions in *Bergey’s Manual* were reliable, and so too are our labels for metabolic traits. If we labeled a species as negative for fermentation in S1 Table, the species likely has not been described as fermentative before.

We found similar agreement between *Bergey’s Manual* and the primary literature for the second trait (acetate production) (Fig 6 and S4 Table). *Bergey’s Manual* reported two species as negative for this trait, even though the primary literature reported them as positive. With few exceptions, our negative labels for acetate production would appear reliable, also.

## Discussion

Microbial metabolism cuts across many fields of biology, yet information on metabolic traits is still hard to access. The information is locked away in text of books and articles. Several attempts have been made to extract this information and make it available in databases [9–11,25,26]. However, the information collected so far, at least for the two traits we investigated, is incomplete. Most attempts to extract information have done so manually, using teams of curators [9–11]. To provide more complete information, a faster method is needed.

We propose neural networks as a fast (and accurate) method to extract information and predict metabolic traits. We provide proof of concept by predicting two metabolic traits for thousands of microbes and with >95% accuracy. This level of performance was high enough to create an accurate phylogenetic tree of these species, and it should be useful for other applications.

The performance of our networks represents an improvement over using other types of machine learning to predict metabolic traits of microbes. Mao et al. [12], for example, predicted traits with a support-vector machine. This approach gave 59% precision and 66% sensitivity when predicting metabolites produced during fermentation. With neural networks, we achieved 93.9% precision and 96.1% sensitivity for a similar prediction (see Fig 2).

Despite the promise of our approach with neural networks, there are still areas that need to be explored. We need to explore, first, sources of species descriptions other than *Bergey’s Manual* [8]. Though *Bergey’s Manual* gave us descriptions for over 7,000 species, this represents only ~1/3 of all species validly published in the literature [19]. We need to explore, second, well our methods work with rare traits. Both metabolic traits we investigated were relatively common (found in over 1,000 species).

Once these uncertainties are resolved, neural networks can be deployed at an even larger scale to predict metabolic traits of microbes. They would enable building of databases of metabolic traits larger than previously imagined. These databases, in turn, will be key to opening up the study of microbial metabolism and bringing it fully into the era of big data.

## Methods

### Preparation of text

To obtain written descriptions of species, articles from *Bergey’s Manual* [8] were downloaded and read into R. Names of species were extracted from the full text, then appropriate sections of the full text were assembled into the description.

Articles in *Bergey’s Manual* [8] were downloaded as html files. This was done using article urls in Browse A-Z page in *Bergey’s Manual* and the download.file() function in R. Only genus-level articles (containing “gbm” in their url) were retained.

The html files were read into R. The full text of each article was then obtained using html_nodes() function and css selectors.

Names of each species were extracted from the full text. For a given article, the genus name was extracted using css selectors. Names of species were then found under the List of Species of the Genus section using the genus name and regular expressions. We reviewed the list of names manually, identified errors, and refined regular expressions (using different expressions to accommodate varying format of articles). Our list also included names of subspecies, biovars, pathovars, and genomospecies, which we treated as equal to species. We used a similar approach (css selectors and regular expressions) to extract other taxonomic ranks and strain IDs.

The full text was parsed to give a written description of each species. The full text typically consisted of 1) Abstract, 2) Further Descriptive Information and other sections about the genus, 3) List of Species of the Genus, and 4) References. These sections were identified using regular expressions. For a given species, we combined text from sections (2) and (3). For (3), we selected only text belonging to the given species, and we excluded text for other species within the genus. This text was selected by using regular expressions for the species name.

### Labeling of metabolic traits

We labeled species as positive or negative for two metabolic traits. Using R and regular expressions, we searched the species descriptions for keywords. For the first trait (fermentative metabolism), the keyword was "ferment". For the second trait (acetate production), the keywords were "ferment" plus "acetate" or "acetic". The regular expression allowed matches not just to the keyword itself, but to any word containing it. For the keyword “ferment”, the words “ferment”, “fermenter”, and “non-fermentative” would all match. When there was a match to the keyword, we read species descriptions in full before labeling the species as positive or negative for the trait. We have experience in reading and labeling species descriptions for these two particular traits [27]. If there was no match, the species was labeled as negative.

### Construction of neural networks

Neural networks were built and trained with TensorFlow [18]. TensorFlow was run in RStudio using the Keras library.

Written description of each species were prepared for input into the network. Sentences matching the keywords were kept, and others were discarded. For the first trait (fermentative metabolism), the keyword was "ferment". At least one sentence had to match "ferment" for any to be kept. For the second trait (acetate production), the keywords were "ferment" plus "acetate" or "acetic". Some sentences were duplicated, and these were discarded. The remaining sentences were joined together and truncated at 25,000 characters. Afterwards, the text was tokenized using the text_tokenizer(), fit_text_tokenizer(), and texts_to_sequences() functions with num_words of 3,000. The tokenized text was then inputted into the network as a list with one element per species. The average number of tokens (words) for the input text was 102 for the first trait and 120 for the second trait.

Labels of metabolic traits were inputted as a vector with one element per species. The elements were 1 (trait positive) or 0 (trait negative).

The networks had architecture as shown in Figs 2 and 3. They were solved with the loss function binary_crossentropy and adam optimizer. The networks were trained with batch size of 32 for 10 epochs. For small amounts of training data, more epochs (up to 40) were needed to minimize the loss function. The amount of training data was as specified in Figs 2 and 3. All data not used for training were used for evaluating predictions.

Predictions were evaluated using accuracy, F1 score, precision, and sensitivity. Accuracy was calculated as (TP+TN)/(TP+TN+FP+FN), where TP = true positive, TN = true negative, FP = false positive, and FN = false negative. F1 score was calculated as TP/[TP+1/2(FP+FN)]. Precision was calculated as TP/(TP+FP). Sensitivity was calculated as (TP)/(TP + FN).

Computational resources for training and prediction were determined using the time package in Ubuntu 20.04 LTS. The resources were run time and maximum memory. Measurements were completed using all six threads of an Intel Core i5-8500T processor and with 16 GiB of RAM.

### Construction of phylogenetic trees

We constructed a phylogenetic tree of genomes belonging to species from *Bergey’s Manual* [8]. The construction followed the general approach of ref. [28,29] and used sequences of 14 ribosomal proteins.

First, we used the strain IDs of each species to find genome sequences. Specifically, we used the strain ID to find a GOLD organism ID [30], GOLD project ID [30], and the IMG/M genome ID (genome sequence) [25] (see S1 Table). Though we could have searched IMG/M directly with the strain ID, this approach was slow. Some strain IDs were generic (e.g., numbers like “238”) and could match multiple GOLD organism IDs. To make matches more specific, we required the species or genus name to match, also. We identified genome IDs for a total of 2,925 species.

Next, we downloaded amino acid sequences of the ribosomal proteins from IMG/M [25]. We did this using KO IDs for the respective genes (S2 Table) along with IMG/M genome IDs. We discarded sequences that were short (<75% of the average length for a given ribosomal protein).

We aligned sequences with Clustal Omega in R [31–33] and then concatenated them. We discarded columns in the alignment with a large number of gaps (95% or more).

We used aligned and concatenated sequences to create a phylogenetic tree. The tree was calculated using maximum likelihood with RAxML [34] on the CIPRES web server [35]. The parameters are listed in S3 Table.

Final analysis and visualization were done in R. The consensus tree and branch lengths were calculated using phytools [36]. The tree was visualized using ggtree [37]. A total of 2,501 species had genomes with protein sequences that could be included in the final tree.

In the full tree, we highlighted branches belonging to species predicted or observed (labeled) to have a metabolic trait. These predictions were made using the convolutional neural network in Fig 2C and training data for 1,000 species. Species part of training data were not highlighted, even if they had the trait. The resulting trees were the predicted or observed trees in Fig 4. We calculated UniFrac distances between these trees using phyloseq [38].

### Completeness of databases reporting metabolic traits

We investigated the completeness of information in three databases: FAPROTAX [11], BacDive [9], and IMG [25]. We did not investigate the IJSEM database [10] because its information has been subsumed by BacDive [9]. We also did not investigate the MACADAM database [26] because its information is in FAPROTAX [11] and IJSEM [10] databases.

For the three databases, we counted the number of microbial species they report as having a fermentative metabolism. For FAPROTAX (v. 1.2.3) [11], we counted species in two ways. First, we used FAPROTAX as a database, counting the number of species in the database packaged with the tool. Only entries containing both genus and species names were counted. Second, we used FAPROTAX as a search tool. We inputted into FAPROTAX the n = 7,021 species from *Bergey’s Manual* used in the current work. This method led to a higher count of species because it uses all of FAPROTAX’s entries, not just those with genus and species names. For BacDive [9], we used Advanced search > Morphology and physiology > Metabolite (utilization). We set Kind of Utilization to “fermentation” and Utilization activity to “+”. For IMG/M [25], genomes with information on metabolism were displayed using Genome Search > Advanced Search Builder > Metabolism. We searched the output for the keyword “ferment” and then read the description in full.

We also counted the number of species the databases reported as producing acetate. For FAPROTAX, we counted no species because no functional group indicated both fermentative metabolism and acetate production. For BacDive, we entered the same settings as for the first trait (fermentative metabolism). Additionally, we set Metabolite (production) to “acetate” and Production to “yes”. For IMG/M [25], we manually searched the output for the keywords “acetate” and “acetic”, then then read the description in full.

### Species descriptions from the primary literature

We compared species descriptions in *Bergey’s Manual* with those from the primary literature for 64 species of bacteria from the rumen. To be included in the comparison, the species had to

- Appear in the List of Prokaryotic names with Standing in Nomenclature [19];
- Have a type strain isolated from the rumen;
- Be described in at least one peer-reviewed journal article;
- Be fermentative;
- Have products of fermentation reported for at least one substrate.

Species were identified from ref. [27], reviews, and individual papers. As before, we treated subspecies as equal to species. The final list of species and information is reported in S4 Table.

## Supporting information

S1 Table Metabolic traits and other information on species from *Bergey’s Manual*. (XLSX)

S2 Table Ribosomal proteins and database IDs searched. (XLSX)

S3 Table Parameters for calculating the phylogenetic tree in RAxML. (XLSX)

S4 Table Information on species of rumen bacteria found in the primary literature. (XLSX)

S1 Fig Few computational resources were required to train neural networks and predict metabolic traits. As Fig 2, except values shown are run time and memory required for training and prediction. Training included tokenization of text.
(PDF)

S2 Fig Performance of neural networks when inputting full text. As Fig 2, except the full text, not just sentences containing keywords, was inputted. Before tokenization, sentences were truncated to 200,000 instead of 25,000 characters. During tokenization, num_words was set to 5,000 instead of 3,000. The average number of tokens (words) for the input text was 5,817, and it was the same for both traits.
(PDF)

S3 Fig Low distances between predicted and observed trees in Fig 4 confirm these trees are similar. For comparison, we calculated distances between random trees and observed trees; these distances are high. We constructed random trees by randomly choosing branches from the tree of all species in Fig 4. We ensured that random and predicted trees had the same number of branches. Values are means ± SEM of five replicates (trees generated by independent trainings of the network). One replicate corresponds to trees shown in Fig 4, and four additional replicates correspond to trees that for brevity are not shown in Fig 4. *P*-values correspond to a *t*-test.
(PDF)
