# Supplementary figures and images for: Using neural networks to mine text and predict metabolic traits for thousands of microbes
Source: PLoS Comput Biol. 2021 Mar 2;17(3):e1008757. doi: 10.1371/journal.pcbi.1008757 (PMC7954334; doi:10.1371/journal.pcbi.1008757)

S1 Figure

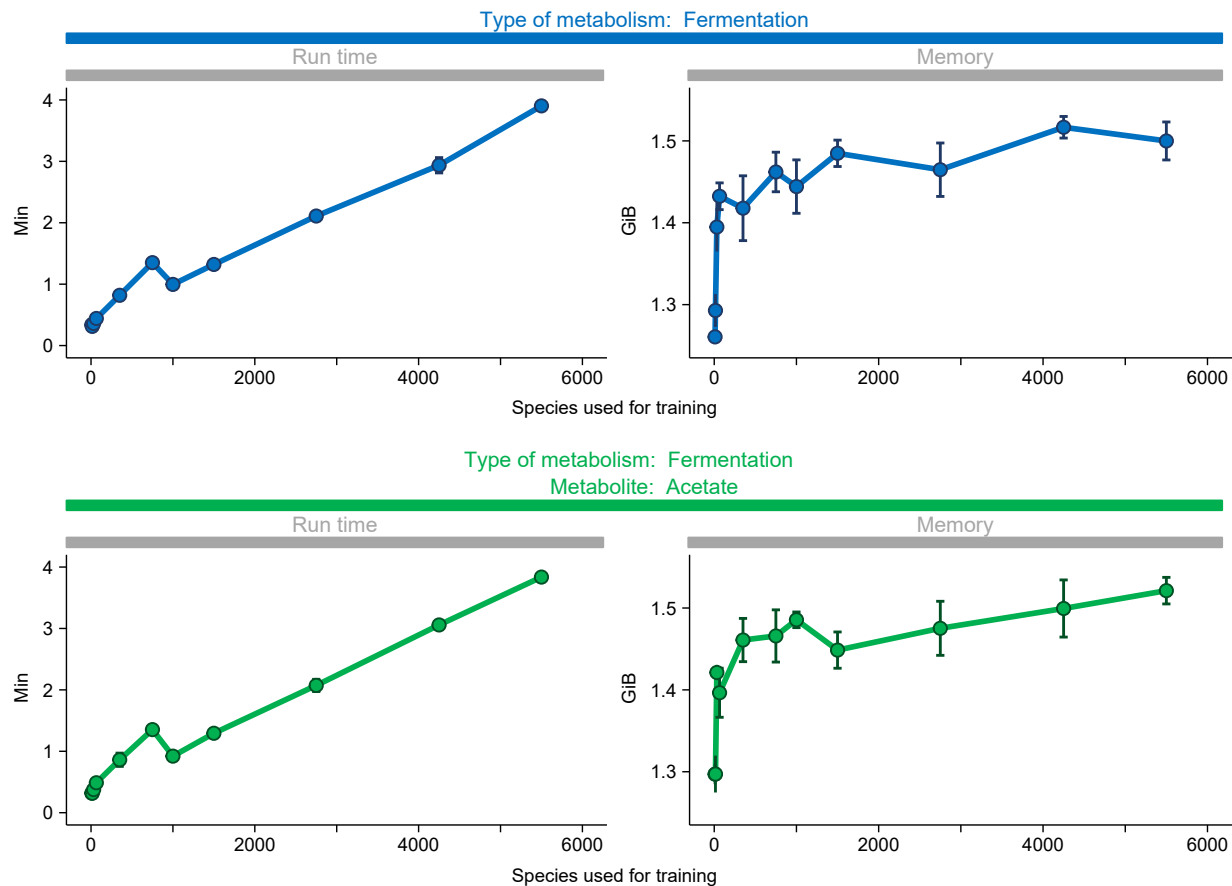

Supplement: S1 Fig — As Fig 2, except values shown are run time and memory required for training and prediction. Training included tokenization of text. (PDF) [file pcbi.1008757.s005.pdf]

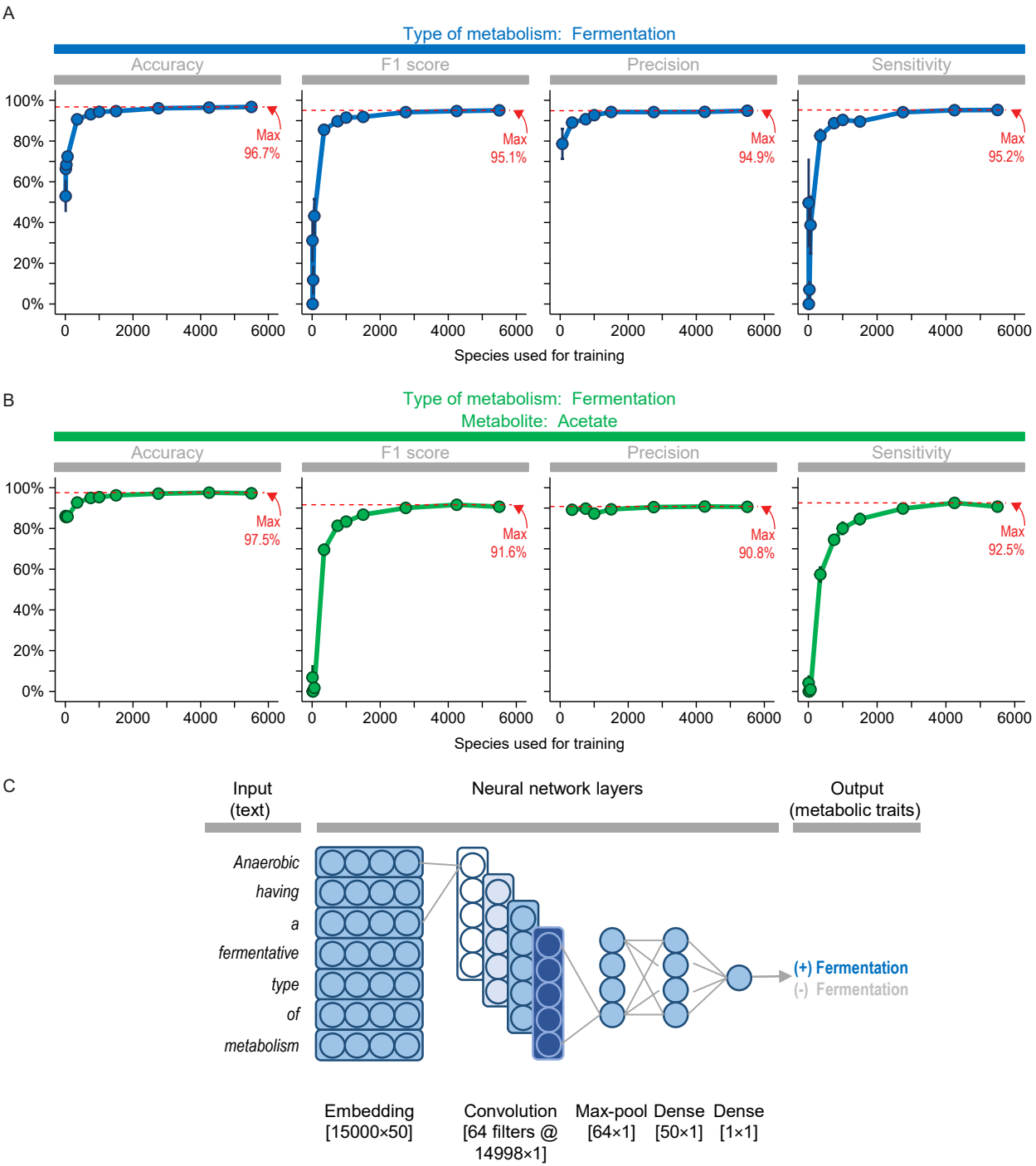

Supplement: S2 Fig — As Fig 2, except the full text, not just sentences containing keywords, was inputted. Before tokenization, sentences were truncated to 200,000 instead of 25,000 characters. During tokenization, num_words was set to 5,000 instead of 3,000. The average number of tokens (words) for the input text was 5,817, and it was the same for both traits. (PDF) [file pcbi.1008757.s006.pdf]

S3 Figure

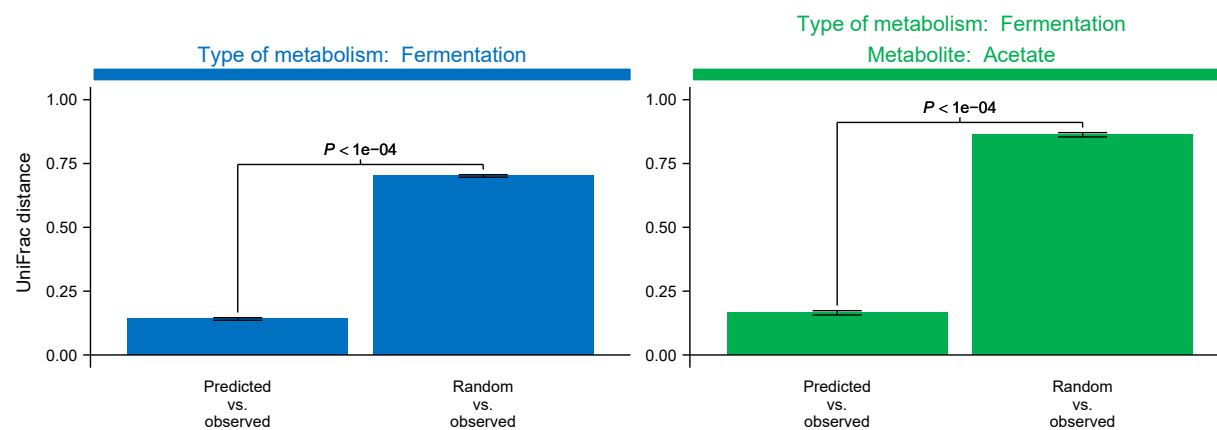

Supplement: S3 Fig — For comparison, we calculated distances between random trees and observed trees; these distances are high. We constructed random trees by randomly choosing branches from the tree of all species in Fig 4. We ensured that random and predicted trees had the same number of branches. Values are means ± SEM of five replicates (trees generated by independent trainings of the network). One replicate corresponds to trees shown in Fig 4, and four additional replicates correspond to trees that for brevity are not shown in Fig 4. P-values correspond to a t-test. (PDF) [file pcbi.1008757.s007.pdf]
